# The 50th Anniversary Conference − Caxambu 2024

**DOI:** 10.1590/0074-02760250061

**Published:** 2026-01-12

**Authors:** Renata Rosito Tonelli, Daniel José Galafasse Lahr, Elvira Maria Saraiva, Angela Hampshire de Carvalho Santos Lopes

**Affiliations:** 1 Universidade Federal de São Paulo, Instituto de Ciências Ambientais, Químicas e Farmacêuticas, Departamento de Ciências Farmacêuticas, Diadema, SP, Brasil; 2 Universidade de São Paulo, Instituto de Biociências, Departamento de Zoologia, São Paulo, SP, Brasil; 3 Universidade Federal do Rio de Janeiro, Instituto de Microbiologia Paulo de Góes, Rio de Janeiro, RJ, Brasil

**Keywords:** Chagas disease, Trypanosoma cruzi, pathogenesis, drug development, public health strategies

## Abstract

Chagas disease (CD), caused by *Trypanosoma* (*Schizotrypanum*) *cruzi*, remains a major global health concern, particularly in Latin America, where millions are at risk. To mark five decades of Chagas research, the Brazilian Society of Protozoology (SBPz) hosted a four-day conference held in Caxambu, Minas Gerais, Brazil, from November 3 to 7, 2024. The meeting brought together world-renowned experts from diverse disciplines whose work has significantly advanced the boundaries of CD studies. Key discussions focused on the parasite’s genetic and metabolic adaptability, with special emphasis on genomic compartmentalisation, RNA processing, and metabolic flexibility essential for survival and pathogenesis. New insights into host-parasite interactions highlighted inflammatory and vascular remodelling processes that drive parasite dissemination and disease progression, especially in cardiac tissue. In the area of drug development, researchers noted treatment limitations, the urgency for novel therapeutic candidates, and ongoing clinical trials assessing alternative regimens of benznidazole (BZN) and nifurtimox (NFX). Progress in biomarker discovery and vaccine development was also discussed as pivotal to improving disease diagnosis, prognosis, and prevention. Beyond laboratory research, the meeting highlighted the importance of science communication and public health engagement. Outreach initiatives and educational exhibitions were showcased as tools to raise awareness and enhance access to disease diagnosis and treatment. Altogether the integration of multidisciplinary approaches from molecular biology to public policy underscores the enduring commitment to combating CD through research, collaboration, and innovation.

Chagas disease (CD), or American trypanosomiasis is a potentially life-threatening parasitic infection caused by the protozoan *Trypanosoma* (*Schizotrypanum*) *cruzi*. While the disease is endemic in 21 Latin American countries, its global relevance has expanded in recent decades due to increased human mobility and migration. According to the World Health Organization (WHO), an estimated 6 to 7 million individuals are currently infected and approximately 75 million people worldwide remain at risk of acquiring the infection.^1^

The primary mode of transmission is vectorial, through contact with the faeces and urine of triatomine bugs harbouring *T. cruzi*. However, oral transmission via ingestion of contaminated food and beverages has become increasingly recognised as a significant epidemiological pathway, particularly in regions where vector control has reduced domiciliary transmission, posing new challenges for disease control. CD manifests in two clinical stages.^2^ The acute phase, which typically lasts between four to eight weeks, is often mild or asymptomatic, thereby complicating early diagnosis. In the absence of treatment, the infection can persist into a chronic phase, during which approximately 30% to 40% of the individuals develop severe cardiomyopathy, gastrointestinal megasyndromes or other systemic complications requiring specialised treatment. Whilst treatment may lower the risk of disease progression, its effectiveness remains inconsistent and not fully understood. Despite the disease’s substantial burden in terms of morbidity, mortality and the socioeconomic impact, diagnostic and treatment coverage remain critically low: fewer than 10% of affected individuals are diagnosed, and only about 1% receive antiparasitic treatment.^3^

Plenary lecture (opening conference)

*Fifteen years of a successful proposal: T. cruz*i *strains should be assigned to one of the discrete typing units* - In this plenary presentation, Bianca Zingales (Universidade de São Paulo, São Paulo, Brazil) provided a comprehensive overview of the genetic and phenotypic diversity of T. cruzi, the causative agent of CD.^4^ Her talk underscored the profound complexity of this protozoan parasite and its relevance to disease outcomes, diagnosis, and treatment strategies.

Dr Zingales highlighted the historical progression of T. cruzi classification systems, culminating in the current consensus framework established in 2009, which recognises six discrete typing units (DTUs), TcI to TcVI, with the subsequent addition of TcBat. She emphasised the significance of genomic studies that have revealed TcV and TcVI as hybrid lineages and documented natural genetic exchange within the species. The presentation further explored how antigenic diversity, particularly in surface proteins, facilitates immune evasion and poses a major obstacle for vaccine development. She discussed the geographical distribution of DTUs, noting that specific lineages tend to predominate in certain regions (*e.g.*, TcI in Central America and the USA; TcII, TcV, and TcVI in Bolivia). Importantly, associations between DTUs and clinical manifestations were addressed, including the prevalence of cardiac complications with TcI and digestive forms with TcII.

She concluded by addressing the challenges posed by this diversity to treatment efforts. Although benznidazole (BZN) and nifurtimox (NFX) remain the standard therapeutic agents, their efficacy appears independent of DTU classification. The speaker called for the establishment of representative, well-characterised strain panels to inform the development of novel diagnostics, vaccines, and therapeutics capable of addressing the broad biological variability of *T. cruzi*.

**Mechanisms of *T. cruzi* pathogenesis**

*Trypanosoma cruzi* is a stealth pathogen that manipulates host tissues to facilitate its survival and dissemination. During infection, the parasite migrates through inflamed tissues, in search of permissive host cells, either macrophages or non-phagocytic cells, where it undergoes intracellular replication.^5^ This process is not silent; intravital microscopy has revealed that microvascular leakage occurs early in infection, triggered by inflammatory signals that open endothelial “floodgates”. This leakage delivers essential nutrients to infected cells, sustaining parasite metabolism, inhibits apoptosis, and contributes to systemic dissemination as host cells rupture and release trypomastigotes. The parasite exploits newly formed microvessels^6^ by engaging endothelial-binding motifs from trans-sialidase (TS) antigens. Research led by Julio Scharfstein’s group (Universidade Federal do Rio de Janeiro - Rio de Janeiro, Brazil) has identified angiogenesis as a hallmark of *T. cruzi* infection, mediated by chymase, a serine protease from mast cells.^6^ This mechanism resembles non-canonical tumor-induced angiogenesis observed in endothelial TLR2-deficient mice, where oxidative lipids promote vascular remodelling. In parallel, extracellular vesicles continuously released by trypomastigotes^7^ are enriched with tGPI-mucins, potent TLR2 activators that may influence inflammatory neovascularisation. The interaction between the mast cell/chymase pathway and the kallikrein-kinin system (KKS) suggests a broader inflammatory mechanism that bridges innate and adaptive immunity in CD.^8^

*Trypanosoma cruzi* infection elicits a strong inflammatory response, characterised by the elevated production of reactive oxygen species (ROS) and nitric oxide (NO). While essential for parasitic control, excessive ROS and NO can cause collateral tissue damage. This is particularly evident in the heart, where *T. cruzi* infection by leads to severe cardiac pathology, including arrhythmias, heart failure, and microvascular abnormalities.^3^ Artur Santos Miranda (Universidade Federal de Minas Gerais, Minas Gerais, Brazil) addressed the roles of oxidative stress and disrupted calcium signalling as key factors influencing *T. cruzi* infectivity and the development of host cardiac Chagasic cardiomyopathy (CCM).His research highlights the enzyme CaMKII, a key regulator of calcium homeostasis and oxidative balance, as a potential therapeutic target. *In vitro* and *ex vivo* studies demonstrate that inhibition of the Ca^2+^/CaM-CaMKII pathway reverses arrhythmic profiles of isolated hearts and isolated left-ventricles cardiomyocytes.^9^ Miranda proposes that dual inhibition CaMKII in both parasite and host could impair parasite replication, reduce inflammation, and prevent cardiac remodelling, ultimately improving disease outcomes.

This multidisciplinary provides new insights into the cellular and molecular mechanisms underpinning *T. cruzi* infection and CCM progression, and points to novel therapeutic strategies that simultaneously target both the parasite and host dysfunction.

Tackling CD

CD remains a critical global health issue. Despite significant progress in recent years, major challenges persist, particularly in drug development and treatment efficacy. A range of complementary approaches now offers a comprehensive view of current advances and future directions in the fight against this neglected tropical disease.^10^

BZN and NFX, the only two drugs approved by the Brazilian Ministry of Health for CD therapy, have been in use for over half a century.^11^ ^,^ ^12^ While these are effective during the acute phase and some chronic cases, they present serious limitations. Treatment regimens are lengthy (60-90 days) and are frequently associated with severe side effects, leading to high rates of discontinuation. Moreover, their efficacy in the chronic phase, where most patients are diagnosed, is limited.^13^ Jadel Müller Kratz (Drugs for Neglected Diseases Initiative, Rio de Janeiro, Brazil) discussed current challenges in developing new, safer and more accessible treatments, for both the acute and chronic stages. His presentation also addressed the translational hurdles involved in drug development, the need to expand access to diagnosis and treatment, the importance of identifying improved biomarkers to assess treatment response and predict disease progression, and the pressing need for greater coordination and investment in the field.^14^

Recent advances in technology have driven progress in drug discovery. Carolina Borsoi Moraes (Universidade de São Paulo, São Paulo, Brazil) highlighted the role, of automated phenotypic screening and sensitive *in vivo* models.^15^ Phenotypic screening remains a cornerstone of antiparasitic drug discovery,^16^ enabling the rapid testing of large compound libraries for antiparasitic activity. This has not only accelerated candidate identification but also provided valuable insights into mechanisms of action. Nevertheless, significant barriers remain, such as the parasite’s ability to persist despite treatment and the continued absence of reliable, clinically validated biomarkers for cure or disease progression.

In this context, Igor Correia de Almeida (University of Texas at El Paso, Texas, USA) presented the TESEO clinical trial (New ThErapies and Biomarkers for ChagaS infEctiOn) that represents a major step forward. With six treatment arms and 450 patients, the TESEO trial is evaluating novel dosing regimens of BZN and NFX, with the aim of identifying strategies that are both safer and more effective.^17^ The trial incorporates the use of molecular biomarkers such as quantitative polymerase chain reaction (qPCR) to improve assessment of treatment response and disease evolution. Importantly these trials underscore the need of tailoring treatments to regional differences in therapeutic responses and advancing biomarker research to predict long-term outcomes.

Ricardo Toshio Fujiwara’s (Universidade Federal de Minas Gerais, Minas Gerais, Brazil) explored the feasibility of a pan-Trypanosomatidae vaccine targeting multiple kinetoplastid parasites, including *T. cruzi*, *Leishmania infantum*, and *Leishmania mexicana*. Capitalising on genetic similarities within the Kinetoplastida class, his team developed a broad-spectrum chimeric polyprotein vaccine incorporating CD8⁺ T cell epitopes conserved across *Leishmania* spp. and *T. cruzi*.^18^ Immunisation with this broad-spectrum vaccine induced specific IgG production, reduced parasite load and decreased inflammation in the colon, fewer degenerated hepatocytes, and increased connective tissue proliferation in the skin lesions. These findings support the concept of a pan-Trypanosomatidae vaccine, paving the way for a new generation capable of targeting diverse infectious agents within this parasite family.

Together, these studies underscore the multifaceted approach required to combat CD, encompassing basic research, drug discovery, translational medicine, clinical trials, and vaccine development. Continued investment and interdisciplinary collaboration are essential to overcome treatment limitations, improve disease management, and advance toward effective control strategies.

**Metabolic adaptability of *T. cruzi***

Several presentations converged on a central theme: the remarkable metabolic adaptability of *T. cruzi*. Each research effort explored distinct aspects of the parasite’s energy metabolism, emphasising emphasising its ability to exploit multiple biochemical pathways to survive in the diverse environments encountered throughout its life cycle. Ariel Mariano Silber (Universidade de São Paulo, São Paulo, Brazil) focused on *T. cruzi’s* ability to utilise histidine as an energy source. His work demonstrated that the parasite efficiently converts this amino acid into ATP through mitochondrial oxidation, highlighting its metabolic flexibility and ability to shift between carbohydrate and amino acid metabolism in response to environmental cues.^19^

Anibal Vercesi (Universidade de Campinas, Sao Paulo, Brazil) explored the intricate interplay between mitochondria and acidocalcisomes in regulating calcium homeostasis. She underscored the importance of mitochondrial calcium ion (Ca²⁺) uptake in buffering cytosolic Ca²⁺ levels and regulating bioenergetics, oxidative phosphorylation, and redox balance. These mechanisms are critical for controlling autophagy and programmed cell death. Central to this process is the mitochondrial Ca²⁺ uniporter (MCU), whose initial identification in trypanosomes contributed to the elucidation of its molecular structure in all eukaryotes.^20^ This research provides new insights into This research provides valuable insights into the composition, function, and physiological role of the MCU in *T. cruzi* and points to new avenues for therapeutic intervention.

*Trypanosoma cruzi* relies on reduced Nicotinamide Adenine Dinucleotide Phosphate (NADPH) as a crucial cofactor for lipid and nucleic acid synthesis, as well as oxidative stress defence. Essential for parasite growth and survival, NADPH is primarily produced through the oxidative branch of the pentose phosphate pathway and enzymes associated with the citric acid cycle.^21^ Arthur Torres Cordeiro’s (Centro Nacional de Pesquisas em Energia e Materiais de Campinas, São Paulo, Brazil) research took a chemical biology approach, exploring how inhibitors targeting key metabolic enzymes can disrupt *T. cruzi* metabolism. He identified inhibitors of enzymes such as glucose-6-phosphate dehydrogenase and malic enzyme, offering promising targets to disrupt the parasite’s metabolic machinery and. viability.

Marcia Cristina Paes (Universidade Estadual do Rio de Janeiro, Rio de Janeiro, Brazil) contributed to understanding the metabolic integration between mitochondria and glycosomes, showing how *T. cruzi* adapts its energy production based on available nutrients. Her findings demonstrated that in glucose-rich environments, *T. cruzi* downregulates mitochondrial activity in favour of succinate fermentation. This metabolic shift enhances survival under variable oxygen conditions, illustrating yet another layer of the parasite’s adaptive capacity.^22^

Collectively, these studies illuminate the extraordinary metabolic plasticity of T. *cruzi*, highlighting its capacity to adjust and fine tune iris energy production pathways, exploit host environments, and ensure survival across different stages and hosts. Whether through amino acid utilisation, organelle interactions, enzyme inhibition, or metabolic shifts, each contribution advances our understanding of the parasite’s physiology and identifies potential metabolic vulnerabilities.

**Key insights into *T. cruzi* biology**

Fundamental aspects of *T. cruzi* biology were explored by six researchers. Maria Carolina Elias (Instituto Butantan, São Paulo, Brazil), discussed the genomic architecture of *T. cruzi* noting its compartmentalised genome. The core regions contain conserved genes, while in disruptive regions are enriched in rapidly evolving multigenic families, potentially enhancing the parasite’s infectivity.^23^ DNA replication origins were found more frequently in four regions, particularly within DGF-1 genes, whereas Core regions appear to lack Orc1Cdc6, a key component of the prereplication complex. Single nucleotide polymorphism (SNP) analysis suggests that DNA replication contributes to genetic variability, thereby promoting adaptability and survival.

Sergio Schenkman (Universidade Federal de São Paulo, São Paulo, Brazil) presented transcriptomic analyses, revealing high transcriptional variability in *T. cruzi*, especially within multigene families. These findings are consistent with previous RNA-seq studies that showed distinct gene expression profiles between virulent and non-virulent T. cruzi strains, with particular emphasis on surface protein genes involved in host interaction.^24^ BZN treatment was shown to alter the transcriptomic profile of intracellular amastigotes, potentially leading to the enrichment of a latent, drug-resistant cell subpopulation. However, issues such as genome variability and host cell RNA contamination highlight the need to optimise of parasite isolation methods to improve transcriptomic study accuracy. Building upon these insights, Irina Afasizheva (University of California, Irvine, USA) explored mitochondrial RNA processing in trypanosomes.^24^ Her team identified the mitochondrial 3′ processome (MPsome), a complex consisting of KRET1 TUTase, KDSS1 3′-5′ exonuclease, and six structural proteins involved in RNA maturation and degradation. The work highlights the role of a DEAD/H-box helicase (KREH3) in processing double-stranded RNA and stabilising guide RNAs. Although focused on T. brucei, these mechanisms may offer important insights for *T.* cruzi research.^25^

José Roberto Sotelo-Silveira (Universidad de la República, Montevideo, Uruguay) investigated the translational machinery of *T. cruzi*, showing that ribosomal protein (RP) mRNA translation is globally repressed during the metacyclic stage, though some RP mRNAs remain active, possibly indicating ribosome specialisation. Additionally, ribosome-associated non-coding RNAs (rancRNAs) derived from snoRNAs and snRNAs appear to modulate translation across different life cycle stages,^26^ highlighting the dynamic regulation of protein synthesis in response environmental changes.

The parasite’s adaptive mechanisms to environmental stresses are illustrated when replicative epimastigotes differentiate into non-proliferative metacyclic trypomastigotes (metacyclogenesis) when nutrients are scarce, and growth is impaired.^27^ Simone Guedes Calderano (Instituto Butantan, São Paulo, Brazil) examined cell cycle dynamics during metacyclogenesis. Most stationary epimastigotes were arrested in the G1 phase (~75%). Expression patterns of six key proteins revealed that cyclin-dependent kinases (CRK1 and CRK2) and DNA replication factors (MCM6 and MCM7) were expressed throughout the cell cycle while cyclin five and Wee1 displayed phase-specific expression peaking at the G1/S transition and Wee1 at the S phase. During metacyclogenesis, CRK3, Wee1, MCM6, and MCM7 expression levels declined, while CRK1 and cyclin five remained active, suggesting a tightly regulated transition that halts proliferation and promotes differentiation.^28^

Studies using cryo-electron microscopy (cryo-EM) and cryo-electron tomography (cryo-ET) have provided unprecedented insights into the parasite’s osmoregulatory system, critical for parasite’s survival. Ingrid Augusto (Universidade Federal do Rio de Janeiro, Rio de Janeiro, Brazil) presented the current knowledge on the crucial role of the contractile vacuole complex (CVC) and acidocalcisome in maintaining *T. cruzi’*s osmotic balance under ionic and osmotic stress conditions.^29^ Acidocalcisome is an electron-dense organelle that stores polyphosphates and cations.^30^ Cryo-ET revealed membrane proteins in the CVC and confirmed the presence of fusion pores, supporting previous findings from frozen-substituted samples. Additionally, acidocalcisomes displayed varying structural organisations and were observed in contact with mitochondria, suggesting possible ion exchange mechanisms.

Once again *T. cruzi* remarkable adaptability of across its life cycle is shown, emphasising the complex interplay between genetic regulation, cell cycle control, and structural dynamics.

Bridging science and society: CD awareness initiatives

For the first time in the 50-year history of the Caxambu meeting, four scientific initiatives on CD were integrated. These activities showcased innovative to raise awareness of CD, its history, and ongoing scientific contributions to its study and control.

Luiz Antonio Botelho Andrade (Universidade Federal Fluminense, Rio de Janeiro, Brazil) presented creative methods to engage schoolchildren in learning about the discovery of CD. His approach incorporates playful and interactive educational tools, such as historical models and stop-motion short films that feature key locations linked to Carlos Chagas’ pioneering work. Developed by the Scientific Audiovisual Laboratory at Universidade Federal Fluminense, this initiative is part of the Integra Chagas Brazil Project (www.integrachagasBrazil.org), and it has been extended to schools in Caxambu with support from SBPz.

Another notable initiative, Expresso Chagas or EC (https://expressochagas.com/), presented by Tania Cremonini de Araújo-Jorge (Instituto Oswaldo Cruz, Rio de Janeiro, Brazil) was social technology project, developed by the Oswaldo Cruz Institute in collaboration with the Rio Chagas Association. EC uses the concept of an ‘imaginary train’ guided by Carlos Chagas to blend science and art as a vehicle for education.^31^ Since its launch, EC has undertaken expeditions in 11 cities, demonstrating technical, social and political viability. The initiative raises public awareness about transmission, prevention, and treatment options, whilst encouraging community engagement in health promotion. To further its reach, EC offers both in-person and virtual training courses for interdisciplinary teams, enabling them to tailor and implement the program in different local settings. Expresso Chagas is aligned with the Brazilian Ministry of Health’s ‘Brazil Saudável’ initiative (https://www.gov.br/saude/pt-br/composicao/saps/Brazil-saudavel), which addresses diseases driven by social determinants.

Added to this there was an engaging interactive game *Escape Room: the lassance enigma* (https://fiocruz.br/escape-room-da-ciencia-Brazileira-1a-parte-descobertas-de-carlos-chagas) developed by Eduardo Caio Torres dos Santos (Instituto Oswaldo Cruz, Rio de Janeiro, Brazil). Inspired by Carlos Chagas’ 1909 expedition to the rural town of Lassance, Minas Gerais, where he identified the unknown disease that led him to make one of the most ground-breaking medical discoveries. The game invites the participants to travel back in time and investigate a medical mystery. The game follows an escape room format, giving players one hour to search for clues, unlock padlocks and solve challenges. Aimed at students and general audiences, the experience fosters curiosity, teamwork and inspires scientific discovery.

Complementing these efforts, the exhibition “A Look at the Past and Present of Chagas Disease” was held in Praça 16 de Setembro, in Caxambu. Conceived by Renata Rosito Tonelli (Universidade Federal de São Paulo, São Paulo, Brazil), Júlia Pinheiro Chagas da Cunha (Instituto Butantan, São Paulo, Brazil), and Maisa Splendore Della Casa (Instituto Butantan, São Paulo, Brazil), the exhibit featured historical photographs and documents from the collection of the Museu de Saúde Pública do Emilio Ribas (https://parquedaciencia.butantan.gov.br/programacao/atracoes/museu-de-saude-publica-emilio-ribas), as well as 3D models of *T. cruzi*, kissing bug specimens, and a microscope to promote hands-onlearning The event was supported by the communication team of the Butantan Institute, funded by “*Loccus do Brazil*” (https://www.loccus.com.br) and assisted by graduate student volunteers. Held one day before the annual meeting, the exhibition attracted over 60 visitors of varying age groups and backgrounds, offering them an accessible introduction to a deeper understanding of CD, its causative agent (*T. cruzi*) and its vector from its discovery to the present day.

Together, these outreach efforts underscore the importance of bridging scientific knowledge and public engagement. By integrating education, creativity and community participation, these initiatives strengthen public health awareness and reinforce the value of science addressing neglected disease like CD.

A legacy of scientific excellence: the research contributions of Professor Erney Plessmann de Camargo

As part of the celebrations, a symposium was held to honour the life and legacy of Professor Erney Plessmann de Camargo (é1935 2023), a distinguished scientist whose career significantly shaped parasitology and microbiology in Brazil and beyond. Professor Jeffrey Shaw (Universidade de São Paulo, São Paulo, Brazil) asserted that Professor Erney Camargo was a trailblazer in parasitology and microbiology, pioneering interdisciplinary studies on emerging vector-borne parasitic diseases such as malaria, medical and veterinary trypanosomiasis, leishmaniasis, and bacterial infections like spotted fever.^32^ ^,^ ^33^ ^,^ ^34^ ^,^ ^35^ One of his most well-known contributions to the study of CD research was the introduction in 1964 of a liquid medium (LIT) for the cultivation of the parasite. His work underscored the importance of a One Health approach, integrating molecular epidemiology, phylogenetics, and taxonomy to deepen our understanding of parasite biodiversity, evolutionary relationships, transmission dynamics, and host-vector interactions.^32^ ^,^ ^36^ ^,^ ^37^ ^,^ ^38^ ^,^ ^39^ His molecular studies on trypanosomatids were instrumental in elucidating their genetic diversity, classification, and life cycles ― contributions with direct implications for disease control in human and veterinary medicine.^36^ ^,^ ^40^ ^,^ ^41^ ^,^ ^42^

A tribute by João Marcelo Pereira Alves (Universidade de São Paulo, São Paulo, Brazil), highlighted Professor Camargo’s contributions to the study of Strigomonadinae trypanosomatids.^43^ These protozoan parasites are characterised by their long-standing endosymbiotic relationship with betaproteobacteria, a symbiosis that evolved over 90 million years ago. Professor Camargo played a pivotal role in uncovering the genetic and biochemical foundations of this association, showing how the endosymbionts supply heme, essential amino acids, and vitamins crucial for the host parasite’s survival.^44^ Through the application of microscopy, biochemistry, and genomic sequencing, he and his colleagues demonstrated how the metabolic pathways of host and symbiont are functionally integrated. This ground-breaking research remains foundational to our understanding of host-microbe interactions at the molecular level.^43^ ^,^ ^44^ ^,^ ^45^

In her tribute, Professor Lucile Maria Floeter-Winter (Universidade de São Paulo, São Paulo, Brazil) emphasised that Professor Erney Camargo was more than just a scientist - he was an educator, mentor, and scientific leader. His influence extended beyond his research, inspiring generations of students and colleagues at USP’s Institute of Biomedical Sciences (ICB). His leadership in national scientific institutions, including the Brazilian Academy of Sciences, the Brazilian Society of Protozoology, the Brazilian Society of Tropical Medicine and the National Council for Scientific and Technological Development (CNPq) demonstrated his commitment to advancing science in Brazil. Beyond academia, he was a staunch advocate for public health policy and the strategic translation of scientific knowledge into effective disease control measures.

Professor Erney Camargo received numerous national and international honours in recognition of his extraordinary contributions to protozoology, parasitology, and molecular biology. His scientific legacy endures, not only through his publications and discoveries, but also in the generation of researchers he mentored and inspired. As Professor Floeter-Winter aptly stated, Professor Erney Camargo was a man ahead of his time, always seeking action and directing his ideas toward advancing science and education.

Historical significance of the CD meeting

During the celebrations, Samuel Goldenberg (Fundação Oswaldo Cruz, Curitiba, Brazil) and Walter Colli (Universidade de São Paulo, São Paulo, Brazil) reflected on the historical significance and impact of the Caxambu CD meeting, which began in 1974. They approached the subject from complementary perspectives.

Samuel Goldenberg analysed scientific publications, including master’s dissertations and doctoral theses related to CD and its causative agent, *T. cruzi*, in parallel Walter Colli offered a detailed account of the meeting’s early years, highlighting key milestones from 1974 to 1975. The year 1974 marked the first effort to unite Brazilian protozoologist, with an initial meeting at CNPq in Rio de Janeiro. In 1975 the inaugural annual meeting on basic research in CD, took place at Hotel Glória in Caxambu, Minas Gerais, organised by Zigman Brener ― one of Brazil’s most eminent parasitologists. The scientific and institutional history of protozoology in Brazil was further explored in an article by Goldenberg et al.^46^ which underscore how the 1974 meeting catalysed methodological standardisation, fostered interdisciplinary collaboration, and positioned Brazil as a leader in parasitic disease research.

Over the decades, advances in medicine and biotechnology have reshaped the research agenda in protozoology. While the early years focused on developing and disseminating experimental methods, contemporary research now prioritises molecular investigations of *T. cruzi*, as well as the study of pathophysiological mechanisms, diagnostics, treatment, and prevention of CD (Figure).

**Figure:**
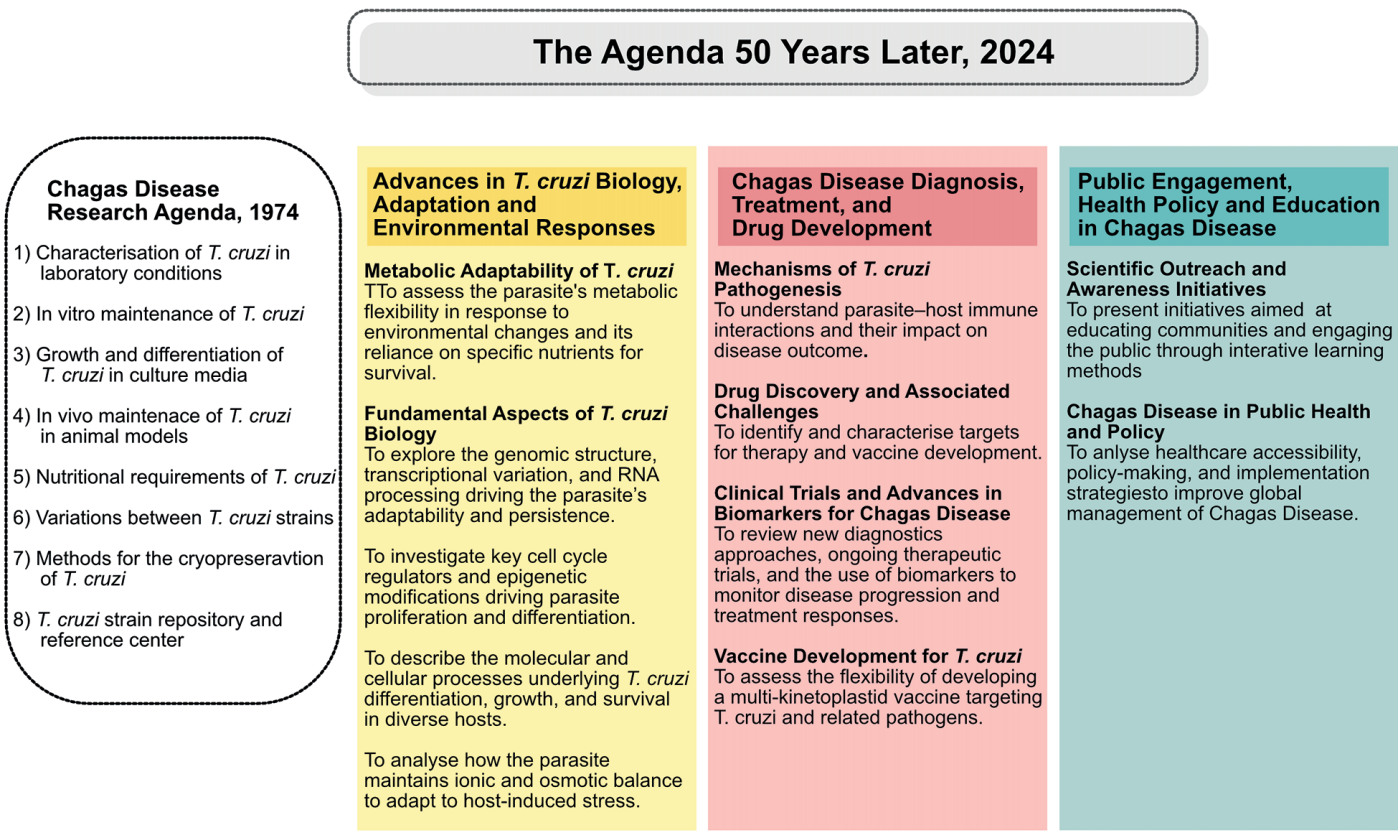
Comparison of the Chagas Disease Research Agenda from the 1970s and 2024. The 1974 agenda focused on fundamental aspects of *Trypanosoma cruzi* biology, including strain characterisation, *in vitro* and *in vivo* maintenance, growth, differentiation, and nutrition. In contrast the 2024 Agenda expands to include environmental adaptation, host-pathogen interactions, novel diagnostics, drug development, vaccine research, and public health initiatives. This evolution highlights the progress in understanding *T. cruzi* and addressing Chagas disease as a global health challenge.

*In conclusion* - In the 1970s, CD posed a significant public health threat in Brazil, particularly in rural areas. At that time, national control programmes were still in their infancy. The agenda for CD in Brazil at that period, according to the 2015 Brazilian Consensus on Chagas Disease^47^ ― developed in collaboration between the Brazilian Society of Tropical Medicine and the Ministry of Health ― the national agenda focused on vector control through insecticide spraying (DDT), ensuring blood transfusion safety, expanding of epidemiological surveillance, promoting research, and introducing early treatment strategies with NFX (launched by Bayer in 1965) and BZN (launched by Roche in 1971).^11^ ^,^ ^12^

Over the past five decades, this Caxambu meeting has evolved into a premier international platform for CD research. It brings together senior experts, early-career researchers, and students to share insights and explore future directions in the fight against CD.

This year’s discussions reaffirmed the multidisciplinary nature of Chagas research, encompassing genomics, parasite metabolism, pathogenesis, drug development, and public health interventions. Highlights included major discoveries in the genetic diversity of *T. cruzi,* new molecular mechanisms of parasite survival, and innovative therapeutic strategies. The incorporation of cutting-edge technologies, such as cryo-electron tomography, multi-omics analyses, and clinical trials, is driving transformative progress in diagnosis, treatments, and vaccine development.

Beyond scientific advancement, the meeting also underscored the importance of community engagement and science communication. Initiatives like Expresso Chagas XXI, educational exhibitions, and interactive learning activities exemplify the researchers’ commitment to bridging the gap between science and society, ensuring that research findings lead to tangible improvements in affected communities.

Looking ahead, CD remains a global health priority. Continued investment, interdisciplinary collaboration, and evidence-based policy action are critical to overcoming persistent barriers in diagnosis, treatment, and prevention. By bringing together scientists, healthcare professionals, and policymakers, the Caxambu meeting continues to serve as a catalyst for innovation, accelerating both scientific discovery and public health impact in the ongoing effort to reduce the burden of CD worldwide.

## References

[1] WHO (2023). Chagas disease (also known as American trypanosomiasis). https://www.who.int/news-room/fact-sheets/detail/chagas-disease-(american-trypanosomiasis.

[2] Velásquez-Ortiz N, Ramírez JD (2020). Understanding the oral transmission of Trypanosoma cruzi as a veterinary and medical foodborne zoonosis. Res Vet Sci.

[3] de Sousa AS, Vermeij D, Ramos AN, Luquetti AO (2024). Chagas disease. Lancet.

[4] Zingales B, Macedo AM (2023). Fifteen years after the definition of Trypanosoma cruzi DTUs: what have we learned?. Life.

[5] Andrade LO, Andrews NW (2005). The Trypanosoma cruzi-host-cell interplay location, invasion, retention. Nat Rev Microbiol.

[6] Vellasco L, Svensjö E, Bulant CA, Blanco PJ, Nogueira F, Domont G (2022). Sheltered in stromal tissue cells, Trypanosoma cruzi orchestrates inflammatory neovascularization via activation of the mast cell chymase pathway. Pathogens.

[7] Gonçalves MF, Umezawa ES, Katzin AM, de Souza W, Alves MJM, Zingales B (1991). Trypanosoma cruzi shedding of surface antigens as membrane vesicles. Exp Parasitol.

[8] Scharfstein J, Andrade D, Svensjö E, Oliveira AC, Nascimento CR (2012). The kallikrein-kinin system in experimental Chagas disease a paradigm to investigate the impact of inflammatory edema on GPCR-mediated pathways of host cell invasion by Trypanosoma cruzi. Front Immunol.

[9] Santos-Miranda A, Costa AD, Joviano-Santos JV, Rhana P, Bruno AS, Rocha P (2021). Inhibition of calcium/calmodulin (Ca2+/CaM) - Calcium/calmodulin-dependent protein kinase II (CaMKII) axis reduces in vitro and ex vivo arrhythmias in experimental Chagas disease. FASEB J.

[10] Chao C, Leone JL, Vigliano CA (2020). Chagas disease historic perspective. Biochim Biophys Acta Mol Basis Dis.

[11] de O Ferreira H (1967). Treatment of Chagas' disease (acute phase) using Bayer 2502. Rev Inst Med Trop São Paulo.

[12] Brener Z (1962). Therapeutic activity and criterion of cure on mice experimentally infected with Trypanosoma cruzi. Rev Inst Med Trop São Paulo.

[13] Kratz JM (2019). Drug discovery for chagas disease a viewpoint. Acta Trop.

[14] Kratz JM, Gonçalves KR, Romera LMD, Moraes CB, Bittencourt-Cunha P, Schenkman S (2022). The translational challenge in Chagas disease drug development. Mem Inst Oswaldo Cruz.

[15] de Sousa NF, Duarte GD, Moraes CB, Barbosa CG, Martin HJ, Muratov NN (2024). In silico and in vitro studies of terpenes from the fabaceae family using the phenotypic screening model against the SARS-CoV-2 virus. Pharmaceutics.

[16] Moffat JG, Vincent F, Lee JA, Eder J, Prunotto M (2017). Opportunities and challenges in phenotypic drug discovery an industry perspective. Nat Rev Drug Discov.

[17] Alonso-Vega C, Urbina JA, Sanz S, Pinazo MJ, Pinto JJ, Gonzalez VR (2021). New chemotherapy regimens and biomarkers for Chagas disease the rationale and design of the TESEO study, an open-label, randomised, prospective, phase-2 clinical trial in the Plurinational State of Bolivia. BMJ Open.

[18] Clímaco MC, de Figueiredo LA, Lucas RC, Pinheiro GRG, Dias Magalhães LM, Oliveira ALG (2023). Development of chimeric protein as a multivalent vaccine for human Kinetoplastid infections Chagas disease and leishmaniasis. Vaccine.

[19] Barisón MJ, Damasceno FS, Mantilla BS, Silber AM (2016). The active transport of histidine and its role in ATP production in Trypanosoma cruzi. J Bioenerg Biomembr.

[20] Docampo R, Vercesi AE, Huang G, Lander N, Chiurillo MA, Bertolini M (2021). Mitochondrial Ca(2+) homeostasis in trypanosomes. Int Rev Cell Mol Biol.

[21] Cordeiro AT (2019). NADPH producing enzymes as promising drug targets for Chagas disease. Curr Med Chem.

[22] Vieira CSD, Aguiar RP, Nogueira NPA, Dos Santos Jr GC.Paes MC (2023). Glucose metabolism sustains heme-induced Trypanosoma cruzi epimastigote growth in vitro. PLoS Negl Trop Dis.

[23] Rosón JN, Vitarelli MO, Costa-Silva HM, Pereira KS, Pires DDS, Lopes LS (2022). H2B V demarcates divergent strand-switch regions, some tDNA loci, and genome compartments in Trypanosoma cruzi and affects parasite differentiation and host cell invasion. PLoS Pathog.

[24] Belew AT, Junqueira C, Rodrigues-Luiz GF, Valente BM, Oliveira AER, Polidoro RB (2017). Comparative transcriptome profiling of virulent and non-virulent Trypanosoma cruzi underlines the role of surface proteins during infection. PLoS Pathog.

[25] Aphasizheva I, Alfonzo J, Carnes J, Cestari I, Cruz-Reyes J, Göringer HU (2020). Lexis and grammar of mitochondrial RNA processing in trypanosomes. Trends Parasitol.

[26] Smircich P, Eastman G, Bispo S, Duhagon MA, Guerra-Slompo EP, Garat B (2015). Ribosome profiling reveals translation control as a key mechanism generating differential gene expression in Trypanosoma cruzi. BMC Genom.

[27] Contreras VT, Salles JM, Thomas N, Morel CM, Goldenberg S (1985). In vitro differentiation of Trypanosoma cruzi under chemically defined conditions. Mol Biochem Parasitol.

[28] Santarossa BA, Mariani É, Corrêa AP, Costa FC, Taylor MC, Kelly JM (2025). Stage-Specific MCM protein expression in Trypanosoma cruzi insights into metacyclogenesis and G1 arrested epimastigotes. Front Cell Infect Microbiol.

[29] Jimenez V, Miranda K, Augusto I (2022). The old and the new about the contractile vacuole of Trypanosoma cruzi. J Eukaryot Microbiol.

[30] Zuma AA, Dos Santos Barrias E, de Souza W (2021). Basic biology of Trypanosoma cruzi. Curr Pharm Des.

[31] Araujo-Jorge TC, Ferreira RR, Rocha RCM, Vieira TM, Costa ND, Santos LL (2021). “Chagas Express XXI”: a new ArtScience social technology for health and science education — A case study in Brazilian endemic areas of Chagas disease with an active search of chronic cases. PLOS Negl Trop Dis.

[32] Espinosa OA, Serrano MG, Camargo EP, Teixeira MMG, Shaw JJ (2018). An appraisal of the taxonomy and nomenclature of trypanosomatids presently classified as Leishmania and Endotrypanum. Parasitology.

[33] Uliana SR, Nelson K, Beverley SM, Camargo EP, Floeter-Winter LM (1994). Discrimination amongst Leishmania by polymerase chain reaction and hybridization with small subunit ribosomal DNA derived oligonucleotides. J Eukaryot Microbiol.

[34] Camargo LM, Ferreira MU, Krieger H, De Camargo EP, Da Silva LP (1994). Unstable hypoendemic malaria in Rondonia (western Amazon region, Brazil): epidemic outbreaks and work-associated incidence in an agro-industrial rural settlement. Am J Trop Med Hyg.

[35] Labruna MB, McBride JW, Bouyer DH, Camargo LM, Camargo EP, Walker DH (2004). Molecular evidence for a spotted fever group Rickettsia species in the tick Amblyomma longirostre in Brazil. J Med Entomol.

[36] Camargo EP (1964). Growth and differentiation in Trypanosoma cruzi. I. Origin of metacyclic trypanosomes in liquid media. Rev Inst Med Trop São Paulo.

[37] Morel C, Chiari E, Camargo EP, Mattei DM, Romanha AJ, Simpson L (1980). Strains and clones of Trypanosoma cruzi can be characterized by pattern of restriction endonuclease products of kinetoplast DNA minicircles. Proc Natl Acad Sci USA.

[38] Alves FP, Durlacher RR, Menezes MJ, Krieger H, Silva LH, Camargo EP (2002). High prevalence of asymptomatic Plasmodium vivax and Plasmodium falciparum infections in native Amazonian populations. Am J Trop Med Hyg.

[39] Labruna MB, Whitworth T, Bouyer DH, McBride J, Camargo LM, Camargo EP (2004). Rickettsia bellii and Rickettsia amblyommii in Amblyomma ticks from the State of Rondônia, Western Amazon, Brazil. J Med Entomol.

[40] Lima L, Espinosa-Álvarez O, Ortiz PA, Trejo-Varón JA, Carranza JC, Pinto CM (2015). Genetic diversity of Trypanosoma cruzi in bats, and multilocus phylogenetic and phylogeographical analyses supporting Tcbat as an independent DTU (discrete typing unit). Acta Trop.

[41] da Silva LH, Camargo EP (1964). Differentiation in the life cycle of trypanosomes. Rev Inst Med Trop São Paulo.

[42] Marcili A, Valente VC, Valente SA, Junqueira AC, da Silva FM, Pinto AY (2009). Trypanosoma cruzi in Brazilian Amazonia: lineages TCI and TCIIa in wild primates, Rhodnius spp. and in humans with Chagas disease associated with oral transmission. Int J Parasitol.

[43] Skalický T, Alves JMP, Morais AC, Režnarová J, Butenko A, Lukeš J (2021). Endosymbiont capture, a repeated process of endosymbiont transfer with replacement in trypanosomatids Angomonas spp.. Pathogens.

[44] Alves JM, Voegtly L, Matveyev AV, Lara AM, da Silva FM, Serrano MG (2011). Identification and phylogenetic analysis of heme synthesis genes in trypanosomatids and their bacterial endosymbionts. PLoS One.

[45] Roitman I, Camargo EP (1985). Endosymbionts of trypanosomatidae. Parasitol Today.

[46] Goldenberg S, Zingales B, Colli W (2025). Basic research on Chagas disease: fifty years of a successful initiative. Acta Trop.

[47] Dias JC, Ramos AN, Gontijo ED, Luquetti A, Shikanai-Yasuda MA, Coura JR (2016). Brazilian Consensus on Chagas Disease, 2015. Epidemiol Serv Saude.

